# A Stress Relief App Intervention for Newly Employed Nursing Staff: Quasi-Experimental Design

**DOI:** 10.2196/15785

**Published:** 2019-12-18

**Authors:** I-Chiu Chang, Wei-Chen Cheng, Wen-Chuan Kung

**Affiliations:** 1 Department of Information Management National Chung Cheng University Chia-Yi Taiwan; 2 Information Technology Office Tainan Municipal An-Nan Hospital Tainan Taiwan; 3 Nursing Department Hsinchu MacKay Memorial Hospital Hsinchu City Taiwan

**Keywords:** nursing staff, occupational stress, mobile app

## Abstract

**Background:**

Most newly employed nurses have limited practical experience, lack problem-solving abilities, and have low resistance to stress, and therefore often opt to resign from the nursing profession.

**Objective:**

This study aimed to assess the effectiveness of a stress relief app (SR_APP) to monitor the stress levels of newly employed nurses.

**Methods:**

We conducted a quasi-experiment to assess changes in stress levels of newly employed nurses at a case hospital, in which the experimental group used the SR_APP and the control group did not. In-depth interviews were conducted to reveal insights regarding their stress. The app usage experiences of experimental group members were assessed via a questionnaire.

**Results:**

All the participants appreciated the experiment and were interested to know more about managing their stress. The experimental group members showed significant differences in heart rate variability scores before and after using the SR_APP, and they reported high levels of intention to use and satisfaction with regard to the SR_APP.

**Conclusions:**

The SR_APP can be effective in helping newly employed nurses to manage their stress.

## Introduction

### Occupational Stress

Work-related stress is a prevalent condition in the nursing profession. A recent literature review showed that studies of nursing stress covered areas from developed to developing countries, departments from general wards to emergency departments, subjects from newly graduated nurses to nurse managers, and topics from the antecedents to the impact of work-related stress [1-11]. Furthermore, significant positive correlation was found between work stress and the demission rate among nursing staff [12]. However, the methods of stress assessment used by those studies are mainly through stress questionnaires and lack objective and quantitative data as references.

Physiological monitoring markers for objective measurements of stress include heart rate, blood pressure, and heart rate variability (HRV). The quantitative analysis of HRV can portray stress indices and circumvent such shortcomings as the intentional concealment of problems during completion of the questionnaire [13]. HRV analysis is widely used as part of the self-adjustment practice for workers in high-stress conditions, students facing examination anxiety, and those in occupations that focus on professional peak performance, such as athletes and artists [14]. This study included HRV as a more objective measurement of stress in nurses. However, various factors can interfere with HRV, including heart rate, age, gender, day and night rhythm, and acute and chronic disease [15]. Therefore, in-depth interviews were conducted to reveal insights regarding their stress.

### Managing Occupational Stress

Stress can be viewed as a personal weakness, and employees are simply obliged to endure workplace stress. A study by Ke et al concludes that high work stress may contribute to nurses overdosing on sedatives, hypnotics, and antipsychotics, and younger nurses becoming more susceptible to drug abuse [16]. Another viewpoint is that stress is both an organizational and personal issue. Therefore, organizations should adopt active and preventive strategies to manage workplace stress. A study by Dewe [17] proposed a 3-level prevention strategy to manage workplace stress. The goal of primary prevention is to eliminate, decrease, or control the number or intensity of stress sources; to modify systems; and to redesign workflow so that the organization’s staff can improve their productivity and increase their motivation. The goal of secondary prevention is to educate and train individuals to identify stress sources and respond to stress effectively. The goal of tertiary prevention is to treat individuals who have already been exposed to stress sources and have experienced physical and mental damage because of it. This study adopted Dewe’s strategy to manage workplace stress of newly employed nurses.

Information technology usage can have a positive effect on performance of organizations, from commercial, manufacturing, to financial ones [18-20]. Similarly, hospitals and clinics have introduced information technology (eg, electronic medical records) with rapid dissemination, processing, and storage capabilities to improve upon the conventional manual workflow and reduce the number of flaws and errors [21-23]. This helps improve the service quality of the entire health care system and increase administrative efficiency. This study considers workplace stress to be a common problem that is both organizational and personal and proposes a stress relief app (SR_APP) in conjunction with the traditional consulting mechanisms for stress management.

## Methods

### Introduction to SR_APP

This study selected a regional teaching hospital in northern Taiwan as its case hospital. This hospital is a religious hospital and places more emphasis on the physical, mental, and spiritual care of their employees. Meanwhile, this hospital is committed to technological advancement with cloud-based medical and nursing information management, which makes this case hospital the leading regional hospital in Taiwan. The SR_APP was created by a project team consisting of 2 representatives from nursing management and administration, 2 representatives from among newly employed nursing staff, 1 clinical psychologist, and 1 external information systems expert. The representatives from nursing management and administration had the job titles of supervisor and head nurse, and each had more than 10 years of experience in counseling and training courses for newly employed staff. The 2 newly employed nurses had been working at the hospital for 1 and 3 months when this study was conducted. The clinical psychologist possesses a professional psychology license and had 9 years of clinical counseling experience at the time of study. The medical information system expert was a professor in information systems from a national university with research interests in the development of nursing systems in hospitals.

**Figure 1:**
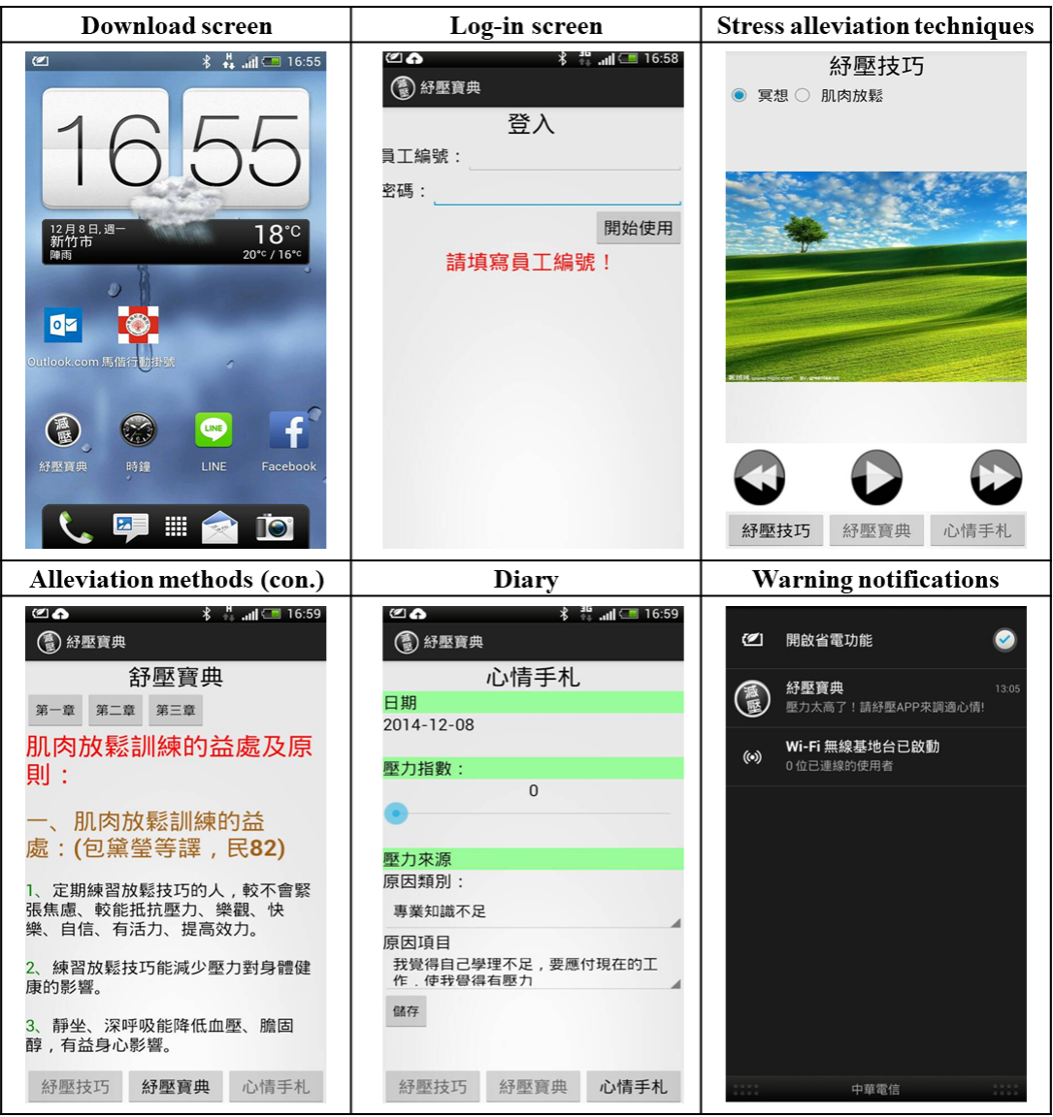
SR-App screenshots.

The design of the SR_APP started with generating users’ requirements from the project team. The information technology staff translated those requirements into system functions and implemented, tested, and revised the app. In total, it took 1.5 months to launch a stable version of the SR_APP. The requirements generated by the project team for the SR_APP were that the system should inform supervisors, staff, and psychologists via email when the staff member’s stress monitoring data exceed the threshold value and when relaxation techniques are used, allow reviewing of historical data, be password protected, have different levels of authorization to access data, and be easy to operate. The information technology staff created the SR_APP according to the aforementioned requirements. The app screenshots are shown in Figure 1.

As the case hospital was granted an International Organization for Standardization specification for an information security management system (ISO 27001) certificate, the research data were stored in the hospital database and accessed under authority control. The nursing manager who was in charge of the newly employed nurses’ learning program had access to the database. Each participant could only access their own data. If they accidently deleted the app, they could reload the app using the Quick Response code on the hospital’s Web page and review the data they had. The data would be kept for 3 years according to the institutional review board (IRB) regulations.

### Research Design

As newly employed nursing staff reported for work at the case hospital, those who met the inclusion criteria (no endocrine or cardiovascular disease, no history of smoking, and not currently on any medication) were invited to participate in the quasi-experiment. Those who agreed to participate were told the aim and methods of the study and were asked to fill out the consent forms and personal background information forms. They were then assigned to the experimental or control group. Ethical approval for the study was obtained from the IRB of MacKay Memorial Hospital—IRB serial number: 14MMHIS045.

**Figure 2:**
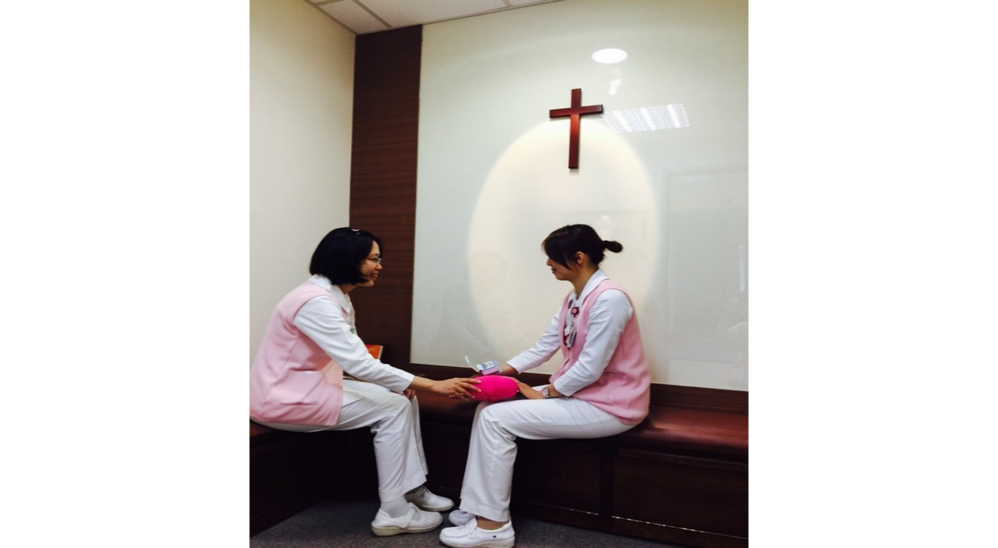
The experiment room setting.

Two times a week, the participants arrived at the experiment room after work, rested for 10 min, and conducted stress assessment using ANSWatch Model TS-0411 as the HRV analyzer. When the stress level of a given participant in the experimental group exceeded the threshold value, an email was sent to that participant’s mobile phone to introduce relaxation techniques. No such intervention was conducted in the control group. If a participant’s stress level was consistently higher than the threshold value, emails were sent to the appropriate supervisor and counselor to arrange a counseling meeting. Participants’ stress levels were continuously measured during the entire time the experiment was conducted. The experiment room setting is shown in Figure 2.

The HRV values of both groups were compared to test the following hypothesis:

H1: Newly employed nursing staff members who use the SR_APP exhibit higher HRV levels than staff members who do not.

After the experiment, in-depth interviews were conducted to allow participants to describe their true feelings about the experiment and the effectiveness of the interventions used in this study. Semistructured, open-ended questions were used to collect the feedback. All the participants agreed to be interviewed after work on the scheduled date. Each interview lasted for approximately 20 to 60 min. A second interview was conducted if there were further questions or additional intact and accurate information had to be obtained. Interview outlines for both groups included questions about their motivation to participate in this study, feedback regarding the experimental process and impacts, and suggestions for improving the study (eg, the experimental process and questionnaire or interview). In addition, the experimental group’s experience of using the SR_APP and their suggestions to improve the SR_APP were assessed using the widely accepted measurement shown in Table 1.

**Table 1:** The items for evaluating the SR_APP.

Variable		Items
**System quality**		
	User-friendly	SR_APP is user-friendly; SR_APP is easy to use
	Interactivity	The SR_APP provides quick feedback; The SR_APP gives me a variety of choices for relaxing
**Information quality**		
	Accuracy	The SR_APP provides accurate information; I am satisfied with the accuracy of the SR_APP
	Immediacy	Information provided by the SR_APP is timely; The SR_APP provides up-to-date information
	Integrity	Information from the SR_APP is valuable; Information from the SR_APP is complete; The SR_APP covers my decompression needs
**Perceived playfulness**		
	Pleasure	When interacting the SR_APP, I am not aware of any noise; Using the SR_APP gives me enjoyment; Using the SR_APP keeps me happy
	User satisfaction	I am satisfied with the “stress alleviation techniques” function in the SR_APP; I am satisfied with the “stress alleviation methods” function in the SR_APP; I am satisfied with the “emotion diary” function in the SR_APP; I am satisfied with all of the functions in this app

The statistics software of Statistical Package for Social Sciences version 19 was used to analyze the collected data. The basic information of the participants was summarized using descriptive statistics. An independent *t* test and 1-way analysis of variance were used to test for significant differences on work stress among different categories. Finally, the Wilcoxon test was used to compare differences between the experimental and control groups before and after intervention measures.

## Results

### Participants Demographic Information

A total of 28 participants were enrolled in this study, of which 21 completed 3 months of stress monitoring (8 in the experimental group and 13 in the control group). The completion rate was 75% (21/28), and 7 participants did not complete the stress monitoring because of the sudden illness of a family member, personal emotional reasons, poor adjustment, and/or resignation from work. The basic information of the participants is shown in Table 2.

Most participants were young females (95%, 20/21) aged between 21 and 25 years (76%, 16/21), who had university or equivalent education, with no work experience (66%, 14/21) and no relevant experience (76%, 16/21). All participants were unmarried, not on medication, nonsmokers, and had no children and no history of acute and chronic disease (eg, hypertension, diabetes, cardiovascular, and endocrine disease).

**Table 2:** Basic information of the participants.

Measure categories		Experimental group (n=8), n (%)	Control group (n=13), n (%)	Total (N=21), n (%)
**Age (years)**				
	<20	2 (25)	3 (23)	5 (23)
	21-25	6 (75)	10 (76)	16 (76)
**Gender**				
	Female	7 (87)	13 (100)	20 (95)
	Male	1 (12)	0 (0)	1 (4.8)
**Education**				
	College	4 (50)	5 (38)	9 (42)
	Bachelor’s	4 (50)	8 (61)	12 (51)
**Ward**				
	Medical Ward	3 (37)	2 (15)	5 (23)
	Surgery Ward	3 (37)	4 (30)	7 (33)
	Emergency	2 (25)	6 (46)	8 (38)
	Outpatient	0 (0)	1 (7)	1 (4)
**Relevant experience^a^**				
	No	5 (62)	11 (84)	16 (76)
	Yes	3 (37)	2 (15)	5 (23)
**Working experience**				
	No	8 (100)	6 (46)	14 (66)
	Yes	0 (0)	7 (53)	7 (33)
**Marriage**				
	Unmarried	8 (100)	13 (100)	21 (100)
**Child**				
	None	8 (100)	13 (100)	21 (100)
**Disease history^b^**				
	None	8 (100)	13 (100)	21 (100)
**Medication**				
	None	8 (100)	13 (100)	21 (100)
**Smoking**				
	None	8 (100)	13 (100)	21 (100)

^a^The prior experience related to the job in current division.

^b^Acute and chronic disease.

### Results of Wilcoxon Test

As the sample size was small, the nonparametric Wilcoxon test was used to compare differences in HRV between the experimental and control groups before and after the intervention measures. The HRV values were ranked, and those ranks were summed for each group. The detailed results shown in Table 3 indicate that before the intervention, members of experimental group had higher stress levels than those of the control group (*P*=.02), and after the intervention, both groups had indifferent HRV scores (*P*=.93).

In other words, the experimental group improved more than the control group. To further explore the differences within the group, the pre- and postaverage scores of HRV for both groups were tested using a paired *t* test. The results are shown in Table 4.

Both groups had significantly higher HRV scores after the intervention, and the experimental group improved about 3 times more than the control group did.

**Table 3:** Wilcoxon test for experimental and control group comparison.

Group		HRV^a^ difference			
		Average rank	Sum of rank	M-H test	*P* value
**Pre**				127.50	.02
	Experimental	16.58	298.50		
	Control	25.90	647.50		
**Post**				221.500	.93
	Experimental	22.19	399.50		
	Control	21.86	546.50		

^a^HRV: heart rate variability.

**Table 4:** Paired t test for experimental and control group heart rate variability score comparison.

Group		HRV^a^ difference		
		Mean (SD)	*t* test (*df*)	*P* value
**Experimental**			–3.661 (7)	<.001
	Pre	27.78 (6.495)		
	Post	73.72 (13.775)		
**Control**			–3.158 (12)	.002
	Pre	32.20 (5.164)		
	Post	47.16 (19.893)		

^a^HRV: heart rate variability.

### Follow-Up Interviews

As aforementioned, certain factors can interfere with the HRV assessment, including heart rate, age, gender, day and night rhythm, and endocrine disease. This study found other factors such as ad hoc assignments, conditions of patients, and critics from clinical preceptors to interfere in the participants’ HRV. Therefore, in-depth interviews were conducted to help understand the insights from the interventions used in this study. The interview data were written into transcripts, and open coding of interview records was conducted based on the subject’s original intention. Meaningful information from the raw data was grouped under similar codes. The in-depth interviews results showed that most newly employed nursing staff valued this experiment as a unique experience and were interested in understanding the changes in their stress levels during the employment process. Therefore, they looked forward to receiving their 3-month test results, regardless of whether they were in the experimental or control groups. Some of the feedback is transcribed below:

I want to know my stress level results.

Although I knew that I would definitely experience stress, I still eagerly want to know test results of my stress level.

Now that I have seen my stress level results, I feel that this is quite interesting.

Initially, I was curious about the final result and felt that it was fun, which was why I agreed to participate in the study!

This is my first job, so I want to know how great is the stress that I will experience.

During each stress assessment, the facilitator observed that the newly employed staff showed fatigue, which was evident in their faces. The facilitator was worried that retaining the subjects for tests would bother them. It turned out that some nurses took advantage of that period of time to emotionally recover from a busy day, though the stress test lasted about 10 min only. Some nurses tried to recall handing over tasks that they had forgotten to perform. Others were so relaxed that they fell asleep. Some nurses persistently complained or even broke down and cried. It seems that this period of time provided an opportunity for newly employed staff to change their mood before returning home.

Staying here for a stress level test enabled me to think about whether I had missed anything.

Apologies, I was too tired and fell asleep during testing!

During this period, I can try to think through what my senior stuff taught me today.

I appreciate this time period to be alone.

I feel like I cannot take this anymore...

I really feel that I am not suitable for this job as I cannot do anything well.

I feel that I will be a burden to my senior staff if I stay.

Newly employed staff experienced work stress everywhere in the workplace, and some stressors were found in the prior studies. Stressors included too many staff names in the department to remember, insufficient staff reporting to work, no common topics for chatting with coworkers, and unfamiliarity with techniques and workflow, as well as receiving new patients, transferring patients, emergency situations, and patients with serious conditions. Additional stress came from mutual disturbances with roommates whose schedules clashed, inconvenient meal times, and lack of parental support.

The department is large with too many senior staff names to remember. I am afraid of identifying the wrong person and feeling embarrassed.

The night shift is fine but there are only two staff members, and I do not know what to chat about and feel awkward throughout the long night.

I am most afraid of receiving new patients as we have to be busy for a while even for one patient.

Every time a new patient arrives, we become very busy.

Patients have stayed for a long period of time, and therefore, we fear having to transfer them.

I am frightened when the patients’ conditions suddenly change.

Although my seniors have taught me some techniques, I am still not familiar with them.

When CPR is performed on the patients, I can only stand aside in a daze and not help as I do not recall what to do.

I am easily awakened by slight noises or light. My roommate also works in shifts, so we often disturb each other.

I cannot eat well because my meal time always changes. I am afraid of disturbing others when I get up and can only lie in a daze.

My mother has been pressuring me to quit, and I do not know what to do.

Most newly employed nursing staff adopted similar methods to relieve stress, such as having a large meal and sleeping off tiredness. Those with stamina left would chat with classmates, friends, and family members regarding work problems and find more courage to continue the nursing profession when they realized that others also encounter similar problems. Only a few nurses would exercise, go shopping, or watch movies to adjust their moods.

While I experienced a lot of stress from working, going home to sleep is my most commonly used method to relieve the stress.

I feel that sleeping leads to a new starting point.

I will have a big meal to get rid of the bad mood from being scolded at work, and feel better.

I feel better after having good food.

Having a big meal after receiving my salary makes me feel better!

When my work is not going well, I will chat with my colleagues who have the same problem and quit thinking that I am not suitable.

We newly employed staff usually get together and complain about the seniors. After we find out that each department has similar problems, we then feel that we are not particularly pitiful.

When I complain to my family members, they will say that it’s the same everywhere.

I will go jogging after work. This enables me to slim down and release some stress.

I will go shopping and watch movies with my friends.

The experimental group had more alternatives by which to relieve their stress, as follows:

In the beginning, I did not use the app. After I experienced more stress, I used it quite often.

For a long while, I would burst out crying in my dorm after work, and using the app helped me fall asleep.

If I could have had this app during my internship, I would enjoy my current job more.

I would follow the steps in the app to relax and felt quite comfortable, so I used it very often.

### Feedback of SR_APP Usage

More information regarding app usage was gathered via the questionnaire. The reliability and validity of the items are as follows. The Cronbach alpha values obtained were .851 for system quality, .833 for information quality, .898 for perceived entertainment, and .844 for usage satisfaction. The overall Cronbach alpha value was .896, which is above .8, indicating that the questionnaire has good reliability. The questionnaire was structured based on a literature review, the study objectives, and the investigators’ practical experience. An expert panel was convened to assess the content validity. The average scores for system quality, information quality, perceived entertainment, and user satisfaction with the SR_APP were 4.22, 3.95, 4.13, and 4.25, respectively, as measured on a 5-point Likert scale where 5 represents highly satisfied and 1 represents highly dissatisfied. In conclusion, the users evaluated the SR_APP system as satisfactory.

## Discussion

### Principal Findings

HRV analysis is widely used in self-adjustment methods for nonmedical workers in high-stress conditions [14]. Endukuru and Tripathi [24] indicated that the components of HRV were sensitive to stress in all healthy individuals. Eller et al [25] found a significant correlation between work stress syndrome and a reduction in the HRV. Some outpatient clinics that specialize in psychiatry and the treatment of emotional stress also use HRV analysis to detect patients’ emotional stress [26,27]. However, applying HRV analysis to the counseling of newly employed nursing staff is rare.

We found that when stress levels exceeded the threshold value, the HRV value decreased, which confirmed the findings of prior studies. The work stress of both groups was reduced significantly. Furthermore, the experimental group’s usage of the app resulted in larger differences in HRV before and after intervention, which indicates that the SR_APP can be used as an additional tool in conjunction with the traditional consulting mechanisms for stress management. An information system is useless if no one uses it. Therefore, the high satisfaction levels and strong usage intention reported by the experimental group after using the SR_APP confirms the value of adding this tool to the conventional stress relaxing methods for newly employed nurses.

A high demission rate not only increases the costs for training new staff but also increases the work burden of existing staff, thereby decreasing the quality of care and possibly affecting patient safety in severe cases [28,29]. The demission rate of newly employed nursing staff of the case hospital during the period of time this study was conducted was 18.84%, as compared with 23.7% in the same period the previous year. By the time this study was conducted, the national average demission rate for hospitals of the same rank was 19.6%. In other words, this shows that the intervention in this study may have also reduced the demission rate of newly employed nursing staff while alleviating employee stress.

### Conclusions

Appropriate technology can help newly employed nursing staff alleviate their work stress when their own stress management skills are poor. The results of this study were inspiring. Not only the case hospital but other chain hospitals would like to adopt the SR_APP. However, the currently used device for measuring the HRV was costly and inefficient. Therefore, the project would continue once the medical engineering department invented an inexpensive and efficient solution for measuring the HRV. By then, the RS-App would be revised accordingly. Although the researchers strove to make the study as rigorous as possible, some limitations could not be overcome because of time and resource constraints. Therefore, several limitations must be considered when making inferences based on the results of this study. First, as the recruitment of the case hospital during the time this study was conducted was not successful, this resulted in a limited number of subjects being invited to participate in the experiment. Second, some subjects were unable to continue participating in the study for personal and family reasons, which led to an uneven number of participants between the experimental and control groups. Third, the subjects were in constant contact with the facilitators during the study period, meeting twice a week for stress measurements, which might have interfered with the test results, especially when some subjects were emotionally unstable because of the excessive stress. To provide timely consolation and care in such cases, the facilitator might find it difficult to maintain a neutral position. Fourth, subjects calling in sick, changing shifts, going on urgent leave, and forgetting to stay after work also made it difficult to monitor their stress with consistent regularity, leading to missing values in the collected data. Finally, this study examined the changes in stress levels of newly employed nursing staff at a case hospital in northern Taiwan. As each hospital has a different culture and management style, caution must be exercised when generalizing the results of this study to other hospitals.

Future studies can perform comparisons and improve the generalizability of our results by extending the sample to different levels or types of hospitals in different areas. There are other factors that may affect the changes in stress levels in newly employed nursing staff and the leadership of clinical preceptors is one of them. It was observed that the clinical preceptors experience great stress when guiding new nurses. Therefore, examining the correlation between stress in preceptors and in newly employed nursing staff may provide a deeper understanding of the work stress for both sides. Meanwhile, some feedback related to the functions of the SR_APP were collected to facilitate future improvements to the system. For example, the interaction function of the current SR_APP will actively show the stress relief techniques only when the user’s stress level exceeds the threshold value. As the reasons why the stress level exceeded the threshold value might be recorded in the participant’s emotion diary, with proper linkage to the diary, the SR_APP can provide appropriate suggestions based on the actual cause. Also suggested for inclusion are additional functions such as the ability to inform the team when 1 member has poor interpersonal relationships in the department and a means by which to manage stress caused by emergency situations encountered by the patient. Finally, more personalized stress relief methods should be added to the app, which will increase the effectiveness with which SR_APP can provide stress relief for newly employed nursing staff.

Nursing staff shortage is a global problem that needs government support. Several successful adoptions of hospital-related information systems have been initiated and supported by the Taiwanese government [30,31]. Thus, on the basis of the findings of this study, the government can encourage hospitals to adopt advanced information technology to provide a friendly work environment for retaining newly employed nursing staff.

## Abbreviations

HRV: heart rate variability
IRB: institutional review board

## References

[1] Frögéli E, Rudman A, Gustavsson P (2019). The relationship between task mastery, role clarity, social acceptance, and stress: an intensive longitudinal study with a sample of newly registered nurses. Int J Nurs Stud.

[2] Liu Y, Aungsuroch Y (2019). Work stress, perceived social support, self-efficacy and burnout among Chinese registered nurses. J Nurs Manag.

[3] Saquib N, Zaghloul MS, Saquib J, Alhomaidan HT, Al-Mohaimeed A, Al-Mazrou A (2019). Association of cumulative job dissatisfaction with depression, anxiety and stress among expatriate nurses in Saudi Arabia. J Nurs Manag.

[4] Pishgooie AH, Atashzadeh-Shoorideh F, Falcó-Pegueroles A, Lotfi Z (2019). Correlation between nursing managers' leadership styles and nurses' job stress and anticipated turnover. J Nurs Manag.

[5] Sonoda Y, Onozuka D, Hagihara A (2018). Factors related to teamwork performance and stress of operating room nurses. J Nurs Manag.

[6] Adriaenssens J, Hamelink A, Bogaert PV (2017). Predictors of occupational stress and well-being in First-Line Nurse Managers: a cross-sectional survey study. Int J Nurs Stud.

[7] Chen CH, Wang J, Yang CS, Fan JY (2016). Nurse practitioner job content and stress effects on anxiety and depressive symptoms, and self-perceived health status. J Nurs Manag.

[8] Johansen ML, Cadmus E (2016). Conflict management style, supportive work environments and the experience of work stress in emergency nurses. J Nurs Manag.

[9] Lavoie S, Talbot LR, Mathieu L, Dallaire C, Dubois M, Courcy F (2016). An exploration of factors associated with post-traumatic stress in ER nurses. J Nurs Manag.

[10] Admi H, Eilon-Moshe Y (2016). Do hospital shift charge nurses from different cultures experience similar stress? An international cross sectional study. Int J Nurs Stud.

[11] Ríos-Risquez MI, García-Izquierdo M (2016). Patient satisfaction, stress and burnout in nursing personnel in emergency departments: a cross-sectional study. Int J Nurs Stud.

[12] Yang H, Lv J, Zhou X, Liu H, Mi B (2017). Validation of work pressure and associated factors influencing hospital nurse turnover: a cross-sectional investigation in Shaanxi Province, China. BMC Health Serv Res.

[13] Jarczok MN, Jarczok M, Mauss D, Koenig J, Li J, Herr RM, Thayer JF (2013). Autonomic nervous system activity and workplace stressors--a systematic review. Neurosci Biobehav Rev.

[14] Williamon A, Aufegger L, Wasley D, Looney D, Mandic DP (2013). Complexity of physiological responses decreases in high-stress musical performance. J R Soc Interface.

[15] Sammito S, Böckelmann I (2016). Factors influencing heart rate variability. ICFJ.

[16] Ke YT, Feng IJ, Hsu CC, Wang JJ, Su SB, Huang CC, Lin HJ (2018). Nurses have a four-fold risk for overdose of sedatives, hypnotics, and antipsychotics than other healthcare providers in Taiwan. PLoS One.

[17] Dewe P (1994). EAPs and stress management: from theory to practice to comprehensiveness. Pers Rev.

[18] Cui T, Ye HJ, Teo HH, Li J (2015). Information technology and open innovation: a strategic alignment perspective. Inform Manag.

[19] Liu H, Wei S, Ke W, Wei KK, Hua Z (2016). The configuration between supply chain integration and information technology competency: a resource orchestration perspective. J Oper Manag.

[20] Tjahjadi B, Soewarno N (2019). The mediating effect of intellectual capital, management accounting information systems, internal process performance, and customer performance. Int J Productivity & Perf Mgmt.

[21] Devaraj S, Ow TT, Kohli R (2013). Examining the impact of information technology and patient flow on healthcare performance: a Theory of Swift and Even Flow (TSEF) perspective. J Oper Manag.

[22] Zardini A, Rossignoli C, Campedelli B (2016). The Impact of the Implementation of the Electronic Medical Record in an Italian University Hospital. Organizational Innovation and Change.

[23] Appiahene P, Missah YM, Najim U (2019). Evaluation of information technology impact on bank’s performance: The Ghanaian experience. Int J Eng Bus Manag.

[24] Endukuru CK, Tripathi S (2016). Evaluation of cardiac responses to stress in healthy individuals-a non invasive evaluation by heart rate variability and stroop test. Int J Sci Res.

[25] Eller NH, Blønd M, Nielsen M, Kristiansen J, Netterstrøm Bo (2011). Effort reward imbalance is associated with vagal withdrawal in Danish public sector employees. Int J Psychophysiol.

[26] Chaudhary R, Haq MM, Shah PS (2015). Changes in heart rate variability in depressed patient. Natl J Integr Res Med.

[27] Green KT, Dennis PA, Neal LC, Hobkirk AL, Hicks TA, Watkins LL, Hayano J, Sherwood A, Calhoun PS, Beckham JC (2016). Exploring the relationship between posttraumatic stress disorder symptoms and momentary heart rate variability. J Psychosom Res.

[28] Dechawatanapaisal D (2018). Nurses' turnover intention: The impact of leader-member exchange, organizational identification and job embeddedness. J Adv Nurs.

[29] Lee HF, Chiang HY, Kuo HT (2019). Relationship between authentic leadership and nurses' intent to leave: the mediating role of work environment and burnout. J Nurs Manag.

[30] Weng SJ, Lai LS, Gotcher D, Wu HH, Xu YY, Yang CW (2016). Cloud image data center for healthcare network in Taiwan. J Med Syst.

[31] Chung MH, Ho CH, Wen HC (2016). Predicting intentions of nurses to adopt patient personal health records: a structural equation modeling approach. Comput Methods Programs Biomed.

